# Freezing–Thawing-Induced Hydrogen Peroxide Formation in PFA Containers

**DOI:** 10.1021/acs.jpclett.6c01551

**Published:** 2026-07-30

**Authors:** Laura Josefine Werner, Alexander Theis, Rutvik Lathia, Tim Peters, Björn Elender, Mischa Bonn

**Affiliations:** † 28309 Max Planck Institute for Chemistry, Hahn-Meitner-Weg 1, 55128 Mainz, Germany; ‡ 9182 Johannes Gutenberg University Mainz, Institute for Atmospheric Physics, Johann-Joachim-Becher-Weg 21, 55128 Mainz, Germany; § 28308 Max Planck Institute for Polymer Research, Ackermannweg 10, 55128 Mainz, Germany

## Abstract

Hydrogen peroxide (H_2_O_2_) and related reactive oxygen species are increasingly reported to form under dark conditions at aqueous interfaces, yet the underlying mechanisms remain debated. Here, we demonstrate that the freezing and thawing of water in fluorinated (PFA) containers generate H_2_O_2_ without external electrical bias or mechanical agitation. H_2_O_2_ formation increases approximately linearly with the number of freezing/thawing cycles and depends strongly on polymer identity, salt concentration, dissolved oxygen, and freezing/thawing temperatures. Perfluoroalkoxy polymer (PFA) tubes produce substantially more H_2_O_2_ than polypropylene (PP) tubes, and the NaCl dependence is qualitatively consistent with previously reported trends associated with contact electrification of aqueous NaCl on fluorinated polymers. Experiments in an ozone-free glovebox demonstrate that dissolved oxygen is required, while rapid freezing and higher thawing temperatures enhance the level of H_2_O_2_ formation. Overall, these findings suggest that freezing/thawing in PFA-based systems may represent a previously underappreciated pathway for H_2_O_2_ formation with potential implications for interpreting low-concentration ROS measurements and understanding interfacial redox processes in freezing environments.

## Graphical abstract

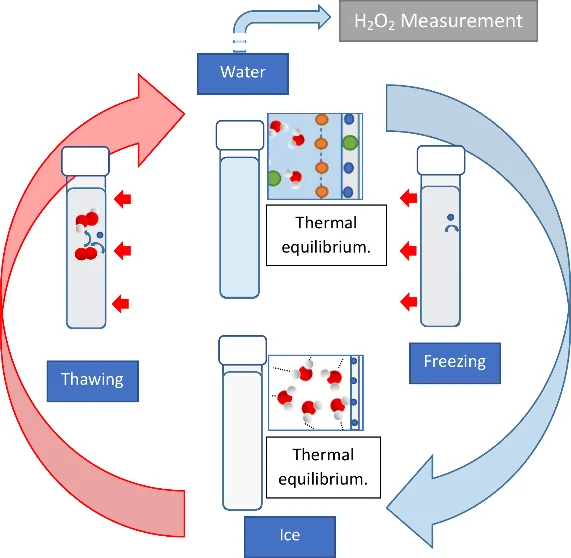

Hydrogen peroxide (H_2_O_2_) and related reactive oxygen species (ROS) are central to atmospheric chemistry,^1^ other environmental redox processes,^2^ and many laboratory and industrial reactions.^3^ Recent work has revealed that H_2_O_2_ can be generated spontaneously at aqueous interfaces, including air–water,^4–8^ ice–water,^9^ and water–solid boundaries,^10–13^ even in the absence of light or added reagents. These findings have triggered intense debate about the underlying driving forces, ranging from strong interfacial electric fields^4–9^ to mechanochemical^11–13^ and alternative explanations involving liquid–solid interfaces and dissolved oxygen,^10,14,15^ and raised concerns about unnoticed ROS sources in both environmental systems and common experimental protocols.

At air–water and ice–water interfaces, several studies have proposed that large interfacial electric fields are sufficient to polarize and activate water molecules, leading to OH radical and H_2_O_2_ formation,^4–9^ whereas other work has argued that observed products can instead originate from liquid–solid interfaces, dissolved gases, or experimental artifacts.^10,14,15^

Parallel to this discussion, contact electrification (CE) between liquids and dielectric solids has emerged as a possible route to interfacial redox chemistry.^11–13,16^ CE describes the generation and accumulation of measurable electrical charge following contact and subsequent separation between two materials.^17^ Despite extensive research, there is still no consensus regarding the microscopic origin of solid–liquid CE.^17^ Proposed microscopic descriptions of contact electrification span electron-transfer-based processes,^18,19^ ion-related interfacial charge redistribution,^20,21^ and electric double-layer-related charge redistribution and polarization effects.^22^ These mechanisms do not necessarily exclude each other and may coexist, with their relative contributions dependent on the system.^18^

Recent experiments on water-droplet sliding electrification further demonstrate that repeated water–solid contact leads to material-specific saturation charge densities, highlighting that substrate chemistry is a key factor governing liquid–solid contact electrification.^23^ Moreover, recent work showed that interfacial electrification is not only material-dependent but also strongly governed by the kinetics of solid–liquid separation. In particular, charge generation at water–hydrophobe interfaces has been shown to be dominated by dewetting dynamics rather than contact formation and to exhibit pronounced history dependence across successive wetting–dewetting cycles.^24^ Together, these findings emphasize that CE is inherently a nonequilibrium, process- and system-dependent phenomenon, in which the interfacial charge state evolves dynamically rather than resetting after each contact event.

The present work does not seek to establish the microscopic origin of the contact electrification. Rather, electron-transfer-based models are used as one possible conceptual framework because they are suggested to provide a direct link between interfacial charge generation and oxygen reduction leading to H_2_O_2_.^11–13,16^ For example, Liu and Bard report that charges generated on Teflon by rubbing with poly­(methyl methacrylate) (PMMA) can participate in redox reactions and propose that these charges are electrons, thereby suggesting that triboelectrically generated electrons can be chemically active in interfacial redox processes.^16^ Wang et al. proposed a mechanism in which CE-induced electron transfer at water–dielectric interfaces oxidizes interfacial water to reactive radicals, while the accumulated electrons subsequently reduce dissolved oxygen, leading to H_2_O_2_ formation under mechanically driven high-energy conditions such as ultrasonication or ball milling.^11–13^

Despite these advances, it remains unclear whether common, low-energy processes, such as the freezing and thawing of bulk water in everyday containers, can themselves provide conditions that may enable interfacial redox chemistry. Freezing of salt solutions generates electric potentials at the ice–liquid interface (Workman–Reynolds effect),^25–28^ which is hypothesized to be a potential driving force for H_2_O_2_ formation,^9^ while fluoropolymers such as PFA are consistently reported among the most tribonegative polymeric materials and exhibit pronounced contact electrification with water.^29^ This behavior has been associated with the high electron affinity of fluorinated surface groups.^29,30^ Lin et al. further reported that fluorinated surfaces exhibit not only larger charge transfer than nonfluorinated surfaces but also a substantially higher fraction of electron transfer relative to ion transfer.^30^ These observations make fluoropolymers, such as PFA, particularly relevant model materials for investigating whether CE may contribute to interfacial redox chemistry and H_2_O_2_ formation.

Recent studies further suggest enhanced CE during solid–liquid phase transitions,^31,32^ yet the relative contributions of CE and freezing-induced electric fields remain unresolved.

If freezing-induced electric fields associated with the Workman–Reynolds effect were a dominant contribution, then the observed H_2_O_2_ formation would be expected to depend primarily on freezing-induced ion separation rather than strongly on the polymer identity. Conversely, a pronounced dependence on the polymer type would point toward an interfacial charge-transfer process.

In this work, freezing and thawing of ultrapure water and aqueous NaCl solutions in fluorinated (PFA) and nonfluorinated (PP) polymer test tubes are used to address this question. By systematically varying freeze–thaw cycles, solution composition, dissolved oxygen, and temperature, and by characterizing liquid–solid CE of the investigated polymers, we probe the relationship between phase transitions, interfacial charge transfer, and H_2_O_2_ formation.

The results reveal a systematic relationship between phase-transition conditions, apparent CE activity, and interfacial H_2_O_2_ formation under otherwise standard laboratory conditions. Comparison with literature reports on CE and Workman–Reynolds potentials is consistent with electron-transfer-based interpretations of contact electrification. In contrast, purely field-driven mechanisms alone do not fully explain the observed trends.

These findings suggest that freeze–thaw cycling in PFA containers may represent an underappreciated source of H_2_O_2_, with potential implications for laboratory ROS measurements and interfacial redox chemistry in freezing environments.

To investigate hydrogen peroxide formation through the freezing and thawing of aqueous systems, various aqueous samples containing either ultrapure water (conductivity ≤ 1 μS/cm) or NaCl solutions of different concentrations were frozen and melted several times in appropriate polymer test tubes. The test tubes were cleaned by sequential rinsing with 1 M NaOH, ethanol, and ultrapure water, then dried with compressed air. The freezing-and-thawing process corresponds to one freezing/thawing cycle (FC). The hydrogen peroxide concentration was then measured. The dependence of hydrogen peroxide concentration on the number of FCs, NaCl concentration, test tube material, gas environment, dissolved oxygen concentration, and freezing and thawing temperatures was investigated. The following describes the methods in more detail.

## Bulk Freezing under Room Conditions

Samples were frozen in an ethanol bath cooled by a cryostat (see Figure S2_1 for a detailed description of the experimental setup) or in liquid nitrogen and thawed in a temperature-controlled water bath. Thermal gradients are expected to develop from the tube wall toward the center. Throughout the freezing and thawing process, analogous samples were stored at room temperature as blank controls to correct the background.

Control experiments showed no systematic loss of H_2_O_2_ during freeze–thaw cycling under the applied conditions observed beyond experimental uncertainty (see SI Section S1 II for details).

## Quantification of H_2_O_2_

H_2_O_2_ concentrations were measured using a fluorescence-based detection system (Aero-Laser AL2021, liquid phase mode, LOD ≥ 0.1 μg L^–1^). Quantification was based on a peroxidase-catalyzed reaction of *p*-hydroxyphenylacetic acid, with and without catalase, to distinguish H_2_O_2_ from organic peroxides.^9^ Calibration was performed using standard H_2_O_2_ solutions (see SI Section S3 for details).

## Glovebox Experiments

Experiments were performed in a glovebox under nitrogen or synthetic air. Ozone levels were monitored using a UV photometric O_3_ analyzer (Dasabi Environmental Corp., model 1108) (see Figure S2_3 for more setup details). To reduce the dissolved oxygen content in water prior to the freezing and thawing cycles, it was distilled under nitrogen flow (≥99.999%) and then quickly transferred to the glovebox (see Figure S2_2). The amount of dissolved oxygen was measured by the portable oxygen meter (DO-230, VWR BRAND), directly after inserting the samples into the test tubes.

## Tilted-Plate Setup

Contact electrification between water and the investigated polymer containers was characterized using a tilted-plate setup^33^ (see SI Section S2 II for setup details).

## H_2_O_2_ Formation Associated with Freezing/Thawing Cycles

To initially test the role of the freezing/thawing process on the H_2_O_2_ formation, a 10^–4^ M NaCl solution and ultrapure water (∼10 mL) were frozen with liquid nitrogen and then melted at ∼20 °C in PFA test tubes (Figure 1). The results are shown in Figure 2.

**Figure 1:**
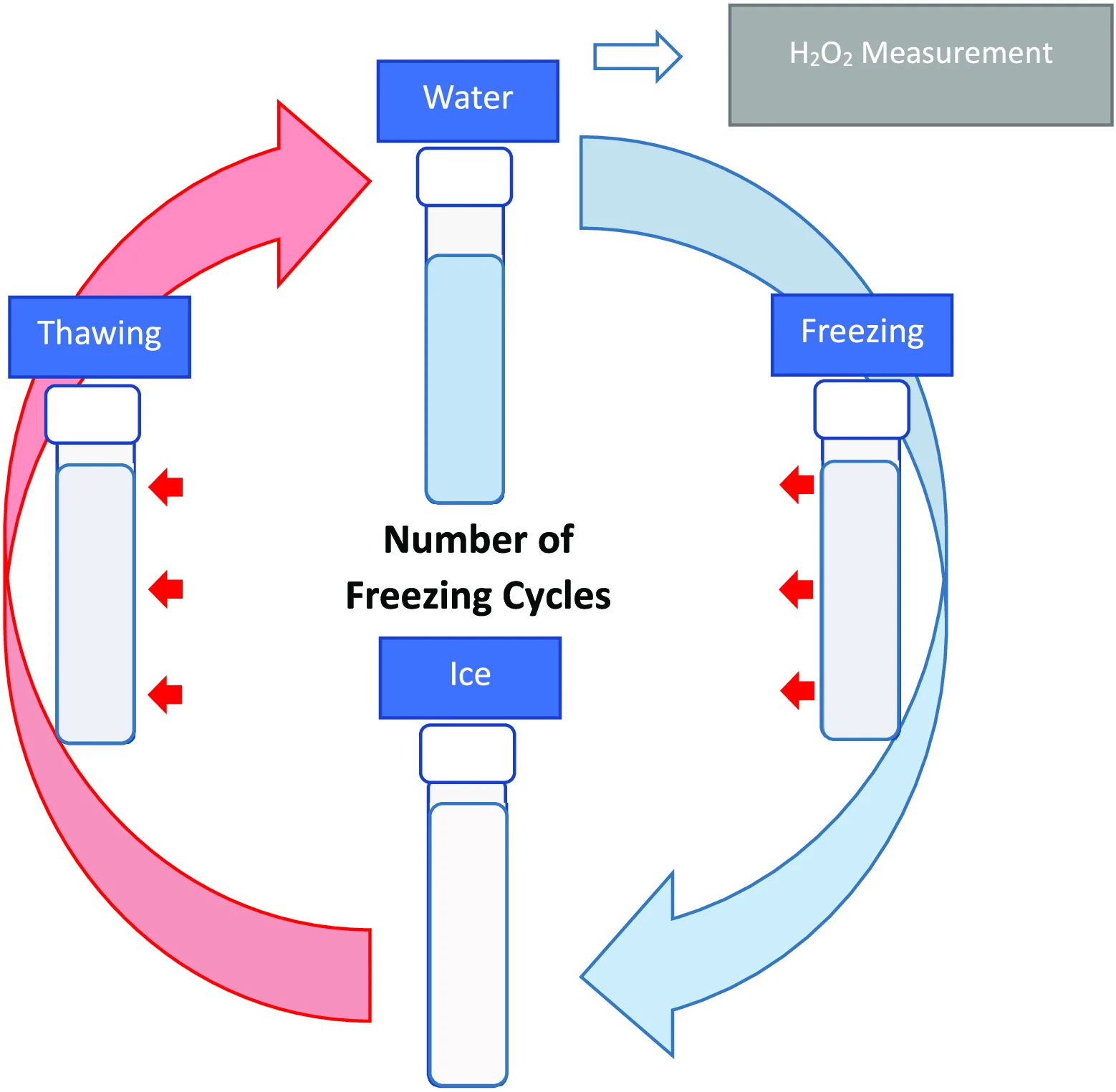
Schematic representation of the freezing/thawing cycles and subsequent H_2_O_2_ measurement.

**Figure 2:**
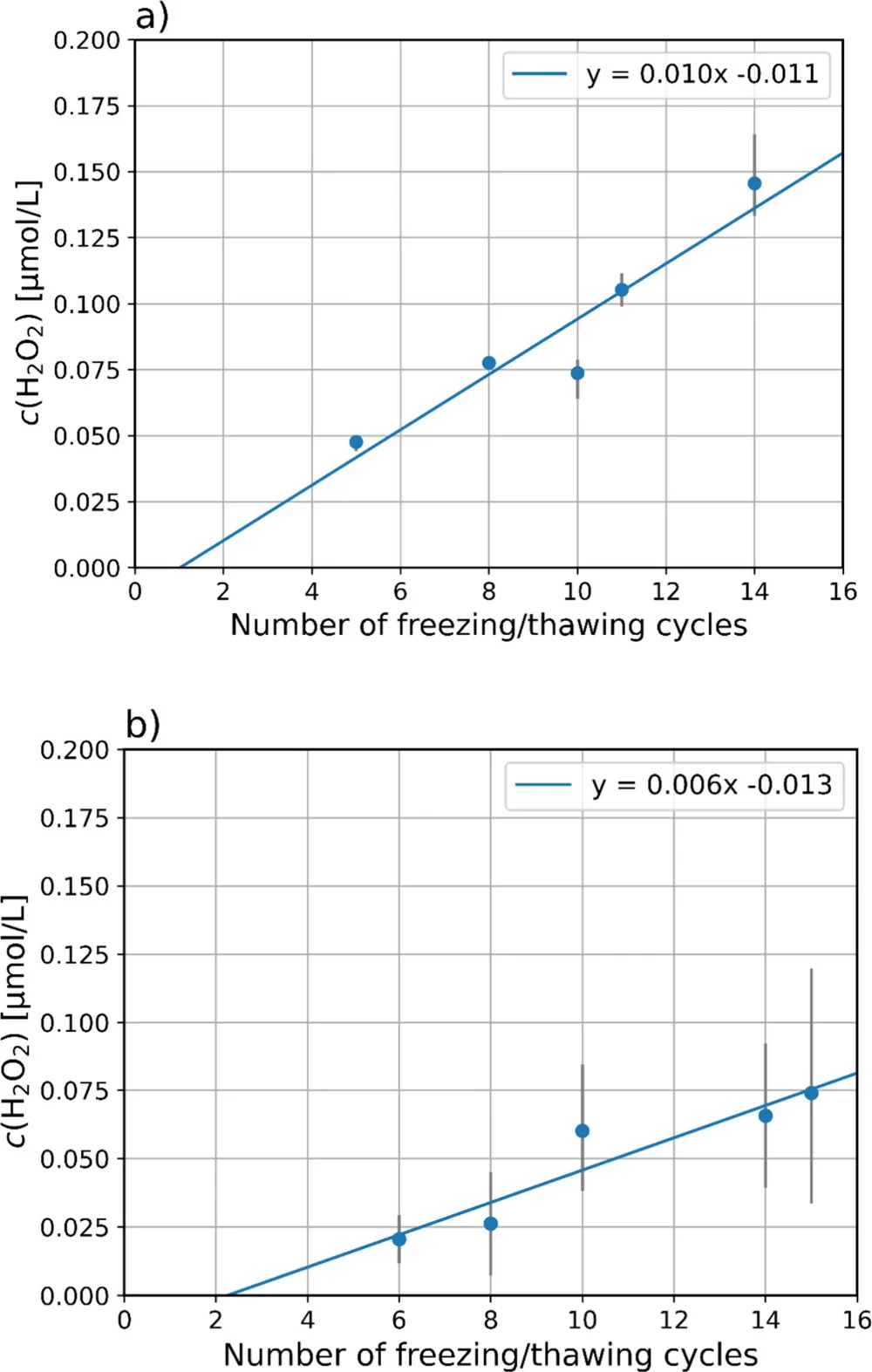
Blank-corrected H_2_O_2_ concentration as a function of the number of freezing/thawing cycles (FC) for (a) an aqueous NaCl solution (10^–4^ M) and (b) ultrapure water, both frozen in PFA test tubes. Freezing was performed in liquid nitrogen, followed by thawing at room temperature (∼20 °C). Errors are represented as the span (deviation of the arithmetic mean from the maximum and minimum values).

The observed linear increase indicates a reproducible accumulation of H_2_O_2_ associated with successive FCs under the experimental conditions. A deviation from linearity at higher cycle numbers cannot be excluded, for example, because of potential saturation effects or changes in interfacial conditions during repeated cycling. The apparent nonzero intercept is likely due to the linear extrapolation of data obtained in a regime where the signal onset is gradual and near the detection limit. While a short initial activation period before the approximately linear increase observed at higher cycle numbers cannot be fully ruled out, no direct evidence of a defined induction phase is observed in the present data.

Such a monotonic increase may reflect a cumulative, cycle-dependent interfacial process. At this stage, this behavior is consistent with both a history-dependent contact electrification mechanism^24^ and repeated ion redistribution effects, such as those associated with the Workman–Reynolds effect,^25–28^ and therefore does not, by itself, allow discrimination between these contributions.

## Salt Concentration Modulates Product Yield: CE vs Workman–Reynolds Effect

The effect of salt concentration on H_2_O_2_ formation during the freezing of aqueous solutions was of particular interest, given the established influence of ions on charge transfer through CE^17–19^ and the reported correlation between ion concentration and hydrogen peroxide formation rates via CEC during ultrasonication of water containing dielectric powder.^12^ Furthermore, the Workman–Reynolds effect,^25^ ion exclusion during ice formation, can generate electric fields at the phase boundary, dependent on ion concentration,^25–27^ which have been suggested as a potential contributor to hydrogen peroxide formation.^9^

The effect of the salt concentration on H_2_O_2_ formation was investigated by freezing and thawing NaCl solutions under different conditions in PFA test tubes. The H_2_O_2_ yields for these samples have been compared with the observed charge of aqueous droplets after contacting a previously discharged PTFE film on a planar conductor, measured by Nie et al.,^19^ and with the WRP measured by Pruppacher et al.^26^ For the samples frozen in liquid nitrogen, only the comparison to Nie et al.^19^ is applied as the WRP has not been defined in this temperature regime. The compared quantities originate from fundamentally different experimental systems and probe different physical observables. This comparison is therefore intended as a qualitative benchmark to assess whether the observed ionic-strength dependence more closely resembles trends reported for contact electrification charge transfer or for the Workman–Reynolds effect. This is not intended to imply a quantitative relationship between these quantities. The results and comparison are given in Figure 3.

**Figure 3:**
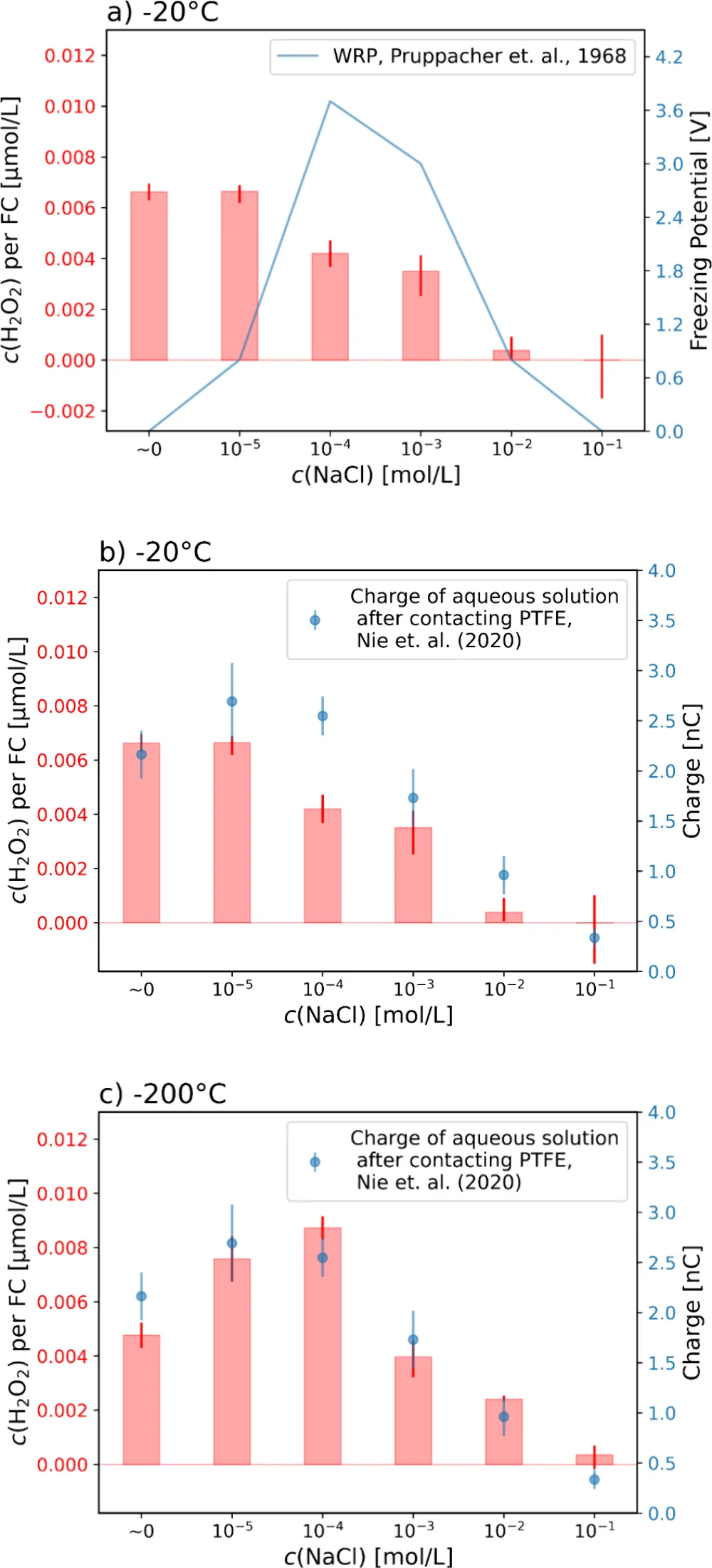
Dependence of the blank-corrected H_2_O_2_ concentration per freezing/thawing cycle on NaCl concentration for samples (a, b) frozen at −20 °C and melted at ∼20 °C and (c) frozen in liquid nitrogen (∼−200 °C) and melted at ∼20 °C. The results are qualitatively compared with (a) the Workman–Reynolds potential (WRP) reported for aqueous NaCl solutions frozen at −15 °C and initiated by local cooling to −20 °C (graphically estimated from Pruppacher et al.,^26^ p. 573, Figure 2; the WRP was reported to be negligibly small for NaCl concentrations >10^–1^ M and <10^–6^ M) and (b, c) with charge measurements obtained for aqueous NaCl solutions (50 μL) after contact with a PTFE membrane (graphically estimated from Nie et al.,^19^ p. 3). The comparison is intended solely to assess qualitative similarities in the ionic-strength dependence and does not imply a quantitative or stoichiometric relationship between H_2_O_2_ formation, transferred charge, and WRP due to the fundamentally different nature of the compared observables and experimental systems. Error bars represent the range (maximum and minimum deviation from the arithmetic mean) for *n* ≤ 3 and the standard error of the mean for *n* > 3. The number *n* is given in the Supporting Information (Table 1).

The H_2_O_2_ yield exhibits a pronounced maximum at low to intermediate NaCl concentrations depending on the freezing conditions. This trend is consistent with reported contact electrification (CE) behavior at water–dielectric interfaces, where charge transfer is enhanced at low to intermediate ionic strength and decreases at higher concentrations.^19^ At low to intermediate ionic strength, the presence of ions may facilitate charge separation and redistribution at the interface, whereas at higher concentrations, electrostatic screening and electric double-layer formation are expected to reduce effective charge transfer, consistent with previous studies on NaCl solutions in contact with PTFE.^19^ In addition, reduced charge transfer at elevated ionic strength has been attributed to faster charge equilibration in solution.^18^

In contrast, the concentration dependence of the Workman–Reynolds potential (WRP) does not reproduce the observed trend in H_2_O_2_ formation, indicating that freezing-induced macroscopic potential differences alone may not explain the observed behavior. In particular, the WRP has been reported to be negligible for ultrapure water,^26^ whereas significant H_2_O_2_ formation is still observed under these conditions. However, it should be noted that the WRP represents a macroscopic measured potential gradient, and direct translation to local field strengths remains nontrivial. Highly localized field spikes, with sufficient magnitudes to drive OH^–^ ionization, have, to the best of our knowledge, not yet been experimentally demonstrated. Nevertheless, contributions from freezing-induced fields cannot be entirely excluded.

The liquid nitrogen freezing regime shows a distinct behavior compared to the −20 °C regime, both in magnitude and in concentration dependence. This suggests that different charge-transfer conditions may prevail under these two freezing regimes. At very rapid freezing rates (liquid nitrogen conditions), limited time for charge relaxation may favor incomplete charge redistribution, while the reduced presence of a mobile interfacial liquid layer and higher local mechanical stresses may enhance charge separation during contact electrification. Under these conditions, the role of supporting ions in stabilizing interfacial charge redistribution may become more pronounced, as reflected by the higher increase in H_2_O_2_ per freezing–thawing cycle for 10^–4^ M NaCl compared to ultrapure water (Figures 1 and 3) and the larger scatter in the ultrapure-water data, represented by the span. At −20 °C, where freezing proceeds over longer time scales, charge-transfer and relaxation processes may be better equilibrated. In this regime, electronic charge transfer during CE may contribute more effectively, whereas dissolved ions primarily introduce electrostatic screening that reduces net charge separation. This difference between freezing regimes is noteworthy because it indicates that the dominant charge-transfer conditions are not invariant with cooling rate and may shift between ion-assisted and more interfacial charge-transfer mechanisms.

Taken together, the observed concentration and temperature dependence are qualitatively consistent with a CE-mediated charge-transfer mechanism for H_2_O_2_ formation, while freezing-induced field effects, as described by the Workman–Reynolds effect alone, do not fully explain the observed behavior. Even under otherwise comparable experimental conditions, a quantitative relationship between transferred charge (or WRP) and H_2_O_2_ yield would not be expected. This is because contact electrification may involve multiple concurrent charge-transfer pathways, with only a subset contributing to interfacial redox chemistry, and because the Workman–Reynolds potential is a macroscopic quantity that is not necessarily directly proportional to local field strengths or potential reaction thresholds. Additionally, alternative influences, including trace contamination and stochastic freezing effects, cannot be ruled out, while small differences in freezing-point depression (≈0.4 °C at the highest salt concentration) and associated volume changes are negligible under the present conditions.

This interpretation remains tentative, and further systematic studies are required to resolve the role of freezing rate and ion-specific effects (e.g., SO_4_^2–^, NO_3_^–^, and Cl^–^).

## Polymer Dependence Further Indicates an Interfacial Mechanism

Building on the previous discussion of the concentration-dependent CE activity and H_2_O_2_ formation, the next question is whether the polymer itself, in particular, its inherent CE propensity, controls the efficiency of the observed redox chemistry. To address this, polypropylene (PP) test tubes were compared with previously used perfluoroalkoxy (PFA) tubes, which were similar in diameter and filling volume. The results for ultrapure water frozen at −20 °C and melted at 20 °C are shown in Figure 4a (see also Figures S1_1 and S1_2 for additional conditions).

**Figure 4:**
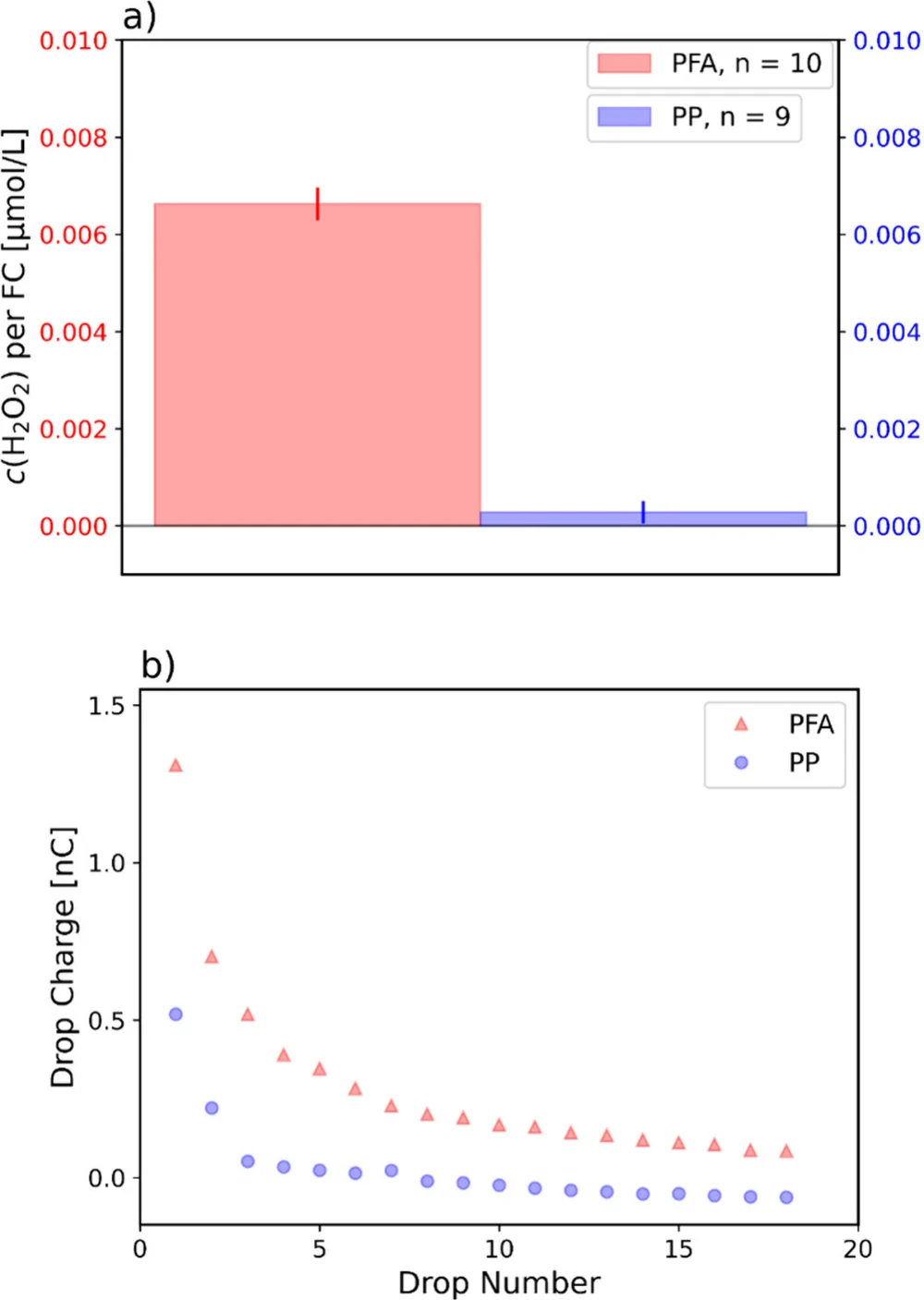
a) Dependence of the H_2_O_2_ concentration per freezing/thawing cycle (FC) on the test tube material for ultrapure water frozen at −20 °C and melted at +20 °C. The data are blank-corrected, and the error bars represent the standard error of the mean. b) Measured droplet charge after sliding Milli-Q water over PP and PFA test tubes using the experimental setup shown in Figure S2_4 of the Supporting Information.

To assess whether the polymer-dependent H_2_O_2_ formation correlates with the contact electrification propensity of the respective polymer, we performed droplet sliding experiments with Milli-Q water on the tilted plate setup^33^ (see SI Section for a detailed setup description). Figure 4b shows the charges of the droplets after sliding over the PP and PFA test tube materials.

The experimental results demonstrate that the H_2_O_2_ yield from freezing and thawing is sensitive to the material of the test tube. Freezing and thawing ultrapure water in PFA tubes leads to a clear, cycle-dependent increase in H_2_O_2_ concentration. In contrast, under identical conditions, no comparable increase is observed in the nonfluorinated PP tubes. The droplet-sliding experiments show a qualitatively consistent trend: For the used PFA and PP test tubes, the charge transfer due to CE is significantly higher in PFA than in PP. For both materials, the droplet charge approaches a plateau after a few passes. Still, in PP, it rapidly decays to values indistinguishable from zero. In contrast, in PFA, it saturates at a finite positive value, suggesting a tendency toward a steady-state-like charge behavior under repeated solid–liquid contact.

Taken together with the data in Figures 4a and S1_1/S1_2, and with reported trends in contact electrification at fluorinated polymer–water interfaces,^29,30^ these findings are consistent with a correlation between the CE propensity of the polymer and measurable H_2_O_2_ formation during freezing and thawing, whereas materials with negligible CE toward water, such as PP under the present conditions, do not show a comparable increase in product formation. In general, contact electrification involves multiple concurrent charge-transfer pathways, which may differ in their ability to generate reactive interfacial species.^17^

The pronounced dependence of H_2_O_2_ formation on polymer identity is not readily explained by a mechanism governed solely by freezing-induced ion separation, but is consistent with a contribution of water–polymer interfacial charge-transfer processes during CE. Nevertheless, contributions from other material-dependent effects or freezing properties cannot fully be excluded.

Moreover, these findings suggest that ambient factors, such as ozone and volatile components of the ethanol bath, do not significantly affect the observed H_2_O_2_ formation, as the experimental procedures were identical. Minor material-dependent factors, such as differences in thermal conductivity, surface roughness, or wettability, may influence freezing dynamics and interfacial conditions but are expected to have a comparatively small effect. If thermal or kinetic effects dominated, then a more gradual variation rather than the observed near-complete suppression of H_2_O_2_ formation would be expected.

It should be noted that this comparison is based on two representative polymers (PFA and PP) chosen for their contrasting interfacial charge-transfer behavior with water and should therefore not be generalized to all fluorinated or nonfluorinated polymer systems.

## Dissolved Oxygen Is Required for H_2_O_2_ Formation

To further elucidate the proposed CE-mediated mechanism, the role of dissolved oxygen as a potential electron acceptor was investigated. Motivated by earlier studies by Mishra and co-workers,^10,15^ which investigated oxygen-dependent H_2_O_2_ formation at solid–liquid interfaces, as well as by Wang, Berbille, and co-workers,^11–13^ who proposed oxygen reduction as a key step in interfacial H_2_O_2_ generation under mechanically perturbed solid–liquid conditions, we investigated whether a similar oxygen dependence is also observed during the considerably lower-energy process of freeze–thaw cycling in PFA containers, in the absence of ultrasonication, ball milling, or externally applied electrical bias.

The role of oxygen was examined using glovebox experiments with controlled gas atmospheres and systematically varied dissolved oxygen levels. Water was either introduced into the test tubes under ambient conditions or first deoxygenated by distillation under high-purity nitrogen (≥99.999%) and subsequently analyzed for its DO content prior to the first freezing/thawing cycle. The dependence of the H_2_O_2_ yield per cycle on DO and gas composition is shown in Figure 5.

**Figure 5:**
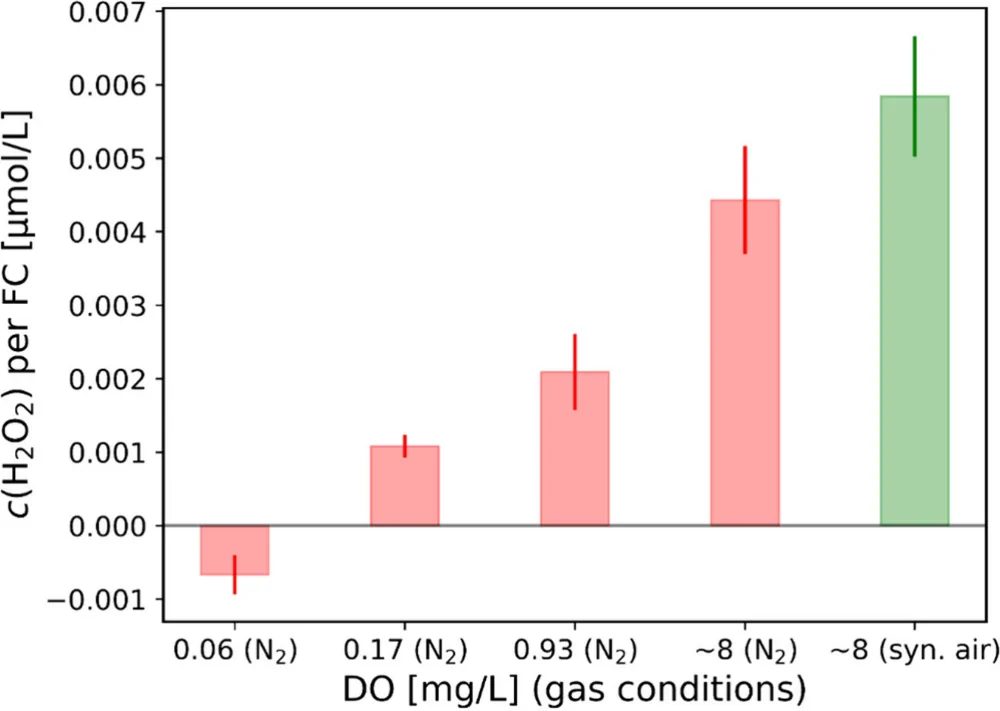
Dependence of the blank-corrected H_2_O_2_ yield per freezing/thawing cycle (FC) for ultrapure water frozen at −20 °C on the surrounding gas atmosphere and dissolved oxygen (DO) concentration (mg/L). Error bars represent the range (deviation of the arithmetic mean from the minimum and maximum values).

The results show that the H_2_O_2_ yield per FC strongly depends on the dissolved oxygen content. As the ozone concentration in the glovebox was ∼0 ppb and the results for ∼8 mg/L DO under either N_2_ or a synthetic air environment show H_2_O_2_ values in the range of those measured under normal room conditions, it can be assumed that the airborne ozone and the surrounding gas do not play a significant role in the observed H_2_O_2_ from bulk freezing. As dissolved oxygen levels decrease, the detectable H_2_O_2_ concentration from freezing/thawing decreases as well. At very low DO concentration (0.06 mg/L), there is less H_2_O_2_ formation detectable than in the corresponding blank samples. As discussed in the SI (Section S1 II), freeze–thaw control experiments indicate that any H_2_O_2_ loss during freezing/thawing cycles is small and close to the experimental uncertainty.

More importantly, the data indicate that dissolved oxygen is an essential requirement for detectable H_2_O_2_ formation under the present conditions. To illustrate a possible mechanistic interpretation consistent with the oxygen dependence, a schematic reaction pathway is shown in Scheme 1.

**Scheme 1:**
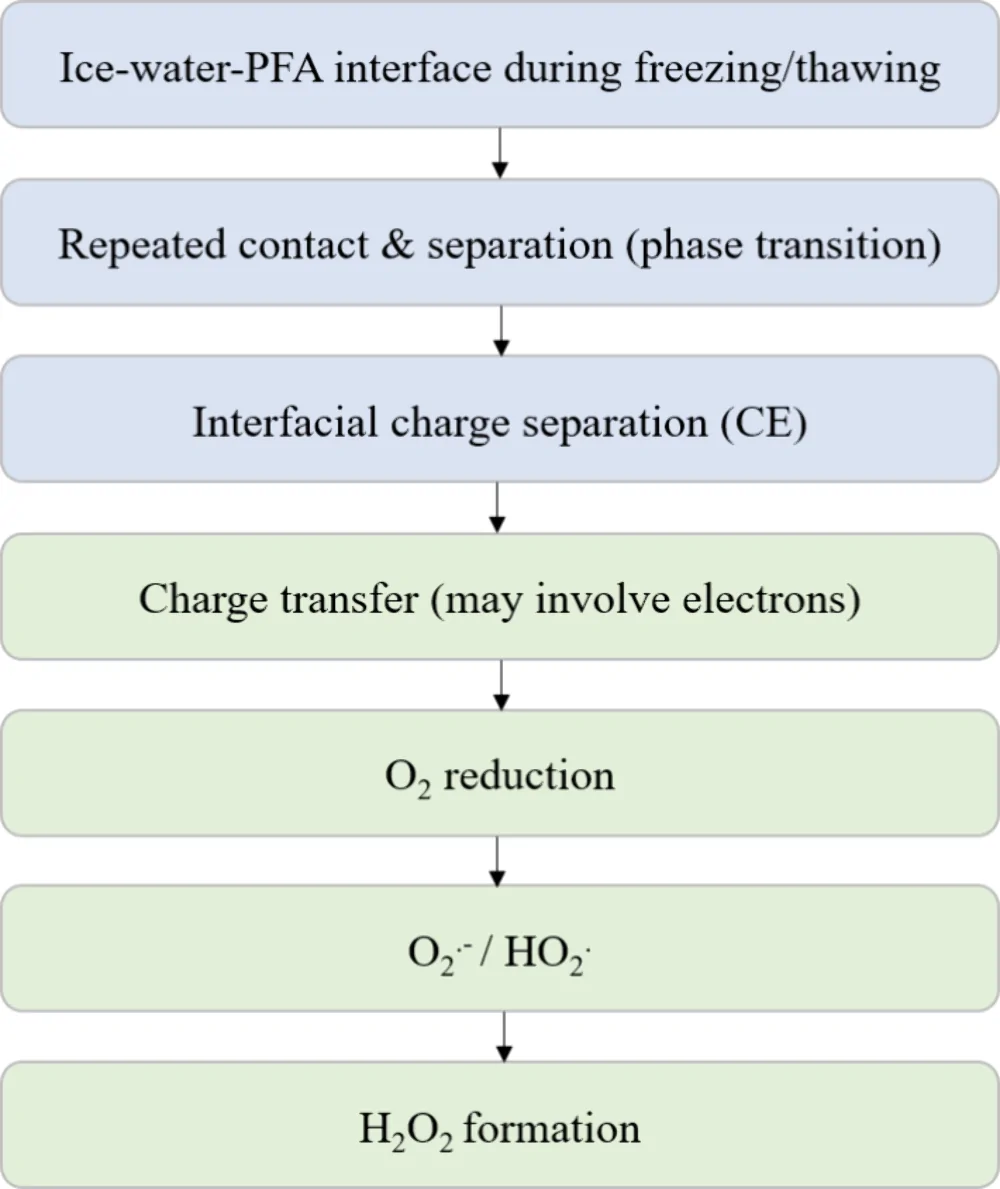
Schematic Illustration of One Possible Interfacial Charge-Transfer Pathway Leading to H_2_O_2_ Formation during Freeze–Thaw Cycling in PFA Containers^a^

a The scheme is intended to illustrate a plausible electron-transfer-based interpretation and does not imply that this represents the unique microscopic mechanism.

In this conceptual framework, repeated phase transitions may induce contact electrification at the ice–water–polymer interface, potentially enabling interfacial charge transfer that involves electrons and subsequent oxygen reduction to reactive intermediates and hydrogen peroxide. The proposed pathway is consistent with previously reported interfacial redox processes suggested to involve contact electrification-driven electron transfer and oxygen reduction at mechanically perturbed solid–liquid interfaces,^11–13^ as well as observations of redox reactions driven by triboelectric charges on insulating surfaces.^16^ While the present data indicate a strong dependence on dissolved oxygen, contributions from alternative reaction pathways or undetected intermediate species cannot be excluded, and the microscopic origin of the transferred charge remains unresolved. Accordingly, Scheme 1 is intended to illustrate only one plausible reaction pathway consistent with the present observations. Under oxygen-limited conditions, the absence of an efficient electron acceptor may lead to negligible net H_2_O_2_ formation, likely because charge separation is not followed by productive redox chemistry.

## Freezing and Thawing Temperatures Affect the H_2_O_2_ Formation

To distinguish the roles of freezing and thawing, we varied both temperatures independently.

Figure S1_5 shows that decreasing the freezing bath temperature (at fixed thawing temperature) increased the H_2_O_2_ yield per cycle, reaching a plateau below about −20 °C for pure water, consistent with its limited supercooling capacity. These observations support the hypothesis that faster freezing, accompanied by stronger volume expansion, pressure buildup, and interfacial friction, may enhance the proposed CE-mediated H_2_O_2_ formation, whereas the absolute ambient temperature appears to be of secondary importance (Figure S1_6). This is consistent with recent studies suggesting that CE depends on interfacial separation kinetics.^24^ In the present system, faster freezing may therefore lead to more rapid interfacial motion and enhanced charge separation. For 10^–4^ M NaCl, H_2_O_2_ formation reached a maximum under the fastest freezing conditions (∼−200 °C), indicating that the electrolyte further enhances H_2_O_2_ formation at extreme freezing rates as previously discussed.

Conversely, when the freezing temperature was held at −20 °C and the thawing temperature was varied, the H_2_O_2_ yield per cycle increased approximately linearly with thawing temperature and followed an Arrhenius-type dependence, yielding an apparent activation energy of about 28 kJ mol^–1^ (5–41 °C), as shown in Figure 6.

**Figure 6:**
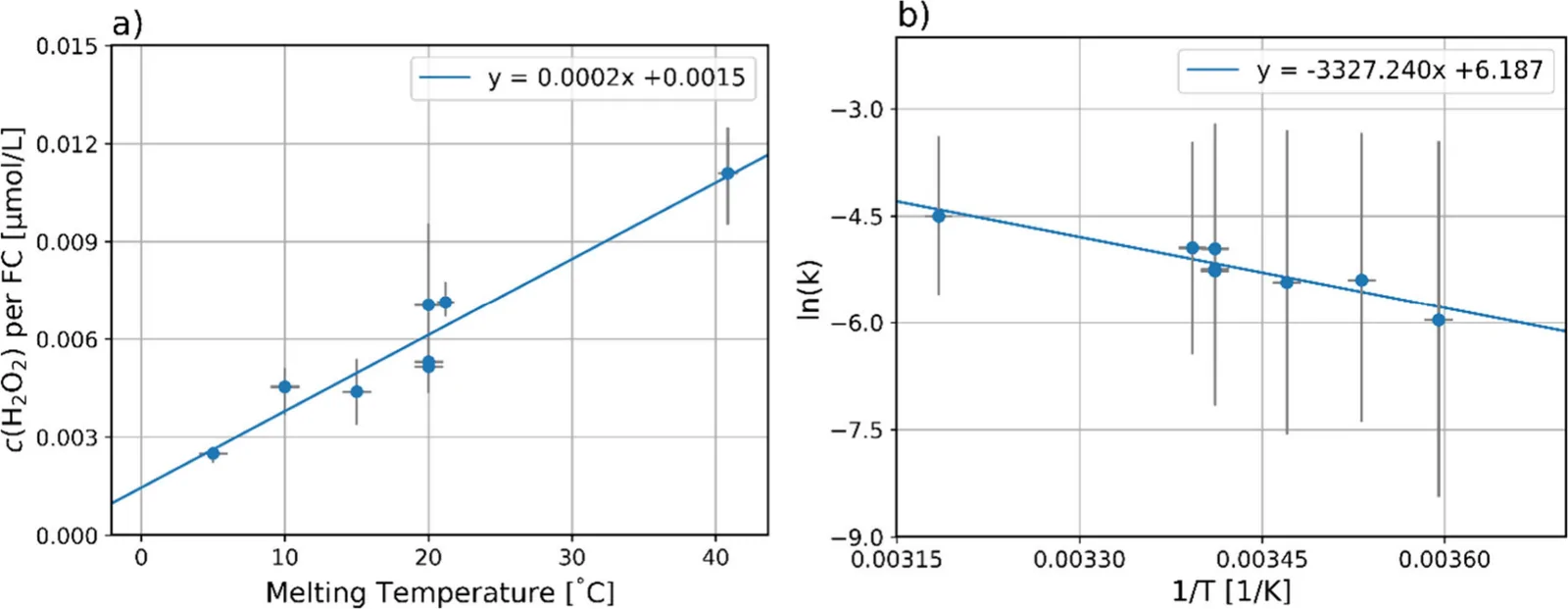
Dependency of the blank-corrected H_2_O_2_ yield per FC for ultrapure water frozen at −20 °C on (a) the thawing temperature and (b) in an Arrhenius plot with k = c­(H_2_O_2_) per FC. Errors in c­(H_2_O_2_) are represented by the range (deviation of the arithmetic mean from the minimum and maximum values). Errors in the thawing temperature are given by the observed cryostat temperature fluctuations.

The Arrhenius-type dependence indicates that one or more thermally activated processes contribute to H_2_O_2_ formation during thawing. Because the system involves coupled phase transitions, oxygen redistribution, interfacial restructuring, and potentially the proposed CE-mediated charge transfer, the apparent activation energy (∼28 kJ mol^–1^) should be regarded as an effective parameter describing the overall process rather than an elementary reaction barrier. As the initial temperature gradient is established across the water–polymer interface, where CE-mediated charge transfer is proposed to occur, this interface likely governs the observed temperature dependence. However, the present experiments do not resolve the individual contributions of the underlying processes, and the observed temperature dependence should therefore be regarded as consistent with, rather than conclusive evidence for, the proposed CE-mediated mechanism.

Freezing and thawing of water in fluorinated (PFA) polymer containers, as reported in this work, provide a reproducible, low-energy pathway associated with hydrogen peroxide formation under conditions that do not require external electrical bias, strong mechanical agitation, or photochemical activation. Taken together, the combined dependence of the H_2_O_2_ yield on salt concentration, polymer type, dissolved oxygen, and freezing/thawing conditions is difficult to reconcile solely with currently reported macroscopic Workman–Reynolds potentials. Instead, the observations are consistent with a contribution of interfacial charge-transfer processes related to contact electrification, in which phase transitions may modulate charge transfer and redistribution at the water–polymer interface, enabling oxygen reduction pathways.

Even though the presented results do not allow for fully disentangling the potential role of contamination or minor electric field contributions from ion separation, they indicate that water-phase transitions in contact with PFA, and potentially with other materials exhibiting strong contact electrification, may act as a sufficient mechanical trigger for interfacial charge transfer and subsequent interfacial redox chemistry in otherwise commonplace laboratory setups.

Beyond establishing a potential new manifestation of CE-mediated redox reactions, the results may have several broader implications. First, they identify routine freezing and storage of aqueous samples in PFA containers as a potential, previously underappreciated source of reactive oxygen species and a possible source of artifacts in sensitive chemical and biological experiments. Second, the observations motivate consideration of whether analogous phase-transition-driven ROS formation processes may occur at interfaces between water and certain highly CE-active materials under environmentally relevant freezing and thawing conditions. Examples may include fluoropolymer surfaces or fluorinated microplastics in cold environments, such as snowpacks, clouds, or cryospheric waters, although whether such systems exhibit similar behavior remains unknown. Natural environments involve substantially greater physicochemical complexity than the simplified laboratory conditions employed here, and the environmental relevance of the observed mechanism remains to be established. Finally, the potential correlation between H_2_O_2_ production and CE activity may motivate the use of phase transitions as a controlled probe of interfacial charge transfer in designed materials.

Future work should aim to directly resolve charge transfer during freezing–thawing, interfacial radical formation, and electron-transfer steps, for example, by employing time-resolved spectroscopy, spin trapping, or isotopic labeling under well-defined freezing and thawing protocols. Extending the materials palette to include polymers and surfaces with systematically varied electronic structure and mechanical properties will help address whether the observations reported in this study can be extended to other interfacial systems and may help establish quantitative structure–activity relationships for phase-transition-driven redox reactions. Moreover, coupling-controlled laboratory measurements with modeling of realistic environmental and experimental scenarios could help assess whether, and under which conditions, analogous processes contribute measurably to atmospheric oxidant formation, microplastic weathering, or laboratory redox chemistry.

## Chemicals

### For Sample Preparation

Water for analysis, EMSURE, Sigma-Aldrich GmbH (conductivity ≤ 1 μS/cm), CAS: 7732-18-5; sodium chloride, Merck KGAa, CAS: 7647-14-5.

### For H_2_O_2_ Quantification

Peroxidase, Carl Roth, CAS: 9003-99-0; potassium hydrogen phthalate, Fischer Scientific, CAS: 877-24-7; hydrogen peroxide 30% for analysis, SUPELCO, CAS: 7722-84-1; hydrochloric acid 37%, for analysis, PanReac Ap, CAS: 7647-01-0; catalase, MP Biomedi, CAS: 9001-05-2; formaldehyde solution ACS Reagent, SIGMA-Aldrich Chemie GmbH, CAS: 50-00-0; EDTA, Carl Roth, CAS: 60-00-4; 4-hydroxyphenylacetic acid, TCI, CAS: 156-38-7; sodium hydroxide (1 M), TCI, CAS: 1310-73-2.

### For Experiments in the Glovebox

Technical nitrogen (>98%), CAS: 7727-37-9; synthetic air, ALPHAGAZ (≥99,999%), CAS: 7782-44-7 + 7727-37-9, nitrogen, ALPHAGAZ (≥99.999%), CAS: 7727-37-9; all gases from AXER24 GmbH & Co. KG.

## Supplementary Material



## Data Availability

The data supporting the findings as well as the corresponding unprocessed raw data of this study are available within the paper and its Supporting Information files.

## Notes

Additional experimental data on the influence of test tube material on H_2_O_2_ formation; freeze–thaw control experiments evaluating potential H_2_O_2_ losses; temperature dependence of H_2_O_2_ yield during freezing; additional experimental setup information for the bulk freezing, tilted-plate, and glovebox experiments; detailed description of the H_2_O_2_ measurement and calibration procedure; and the number of samples used for each experiment presented in Figures 3a–c and S1_5a,b (PDF)
