# Supplementary material for: Freezing–Thawing-Induced Hydrogen Peroxide Formation in PFA Containers
Source: J Phys Chem Lett. 2026 Jul 30;17(32):9372–80. doi: 10.1021/acs.jpclett.6c01551 (PMC13488436; doi:10.1021/acs.jpclett.6c01551)
Supplement: Supplementary file 1 [file jz6c01551_si_001.pdf]

Supplementary Information

Freezing-Thawing-Induced Hydrogen Peroxide Formation in PFA Containers

Laura Josefine Werner[1*], Alexander Theis[1], Rutvik Lathia[3], Tim Peters[1], Björn Elender[2], Mischa Bonn[3*]

[1] Max Planck Institute for Chemistry, Hahn-Meitner-Weg 1, 55128 Mainz, Germany

[2] Johannes Gutenberg University Mainz, Institute for Atmospheric Physics, Johann-Joachim-Becher-Weg 21, 55128 Mainz, Germany

[3] Max Planck Institute for Polymer Research, Ackermannweg 10, 55128 Mainz, Germany

* Corresponding authors:

Laura Josefine Werner
Max Planck Institute for Chemistry
Hahn-Meitner-Weg 1
55128 Mainz
E-mail: l.werner@mpic.de

Mischa Bonn
Max Planck Institute for Polymer Research
Ackermannweg 10
55128 Mainz
E-Mail: bonn@mpip-mainz.mpg.de

Section S1 – Additional Data

I. Role of the Test Tube Material

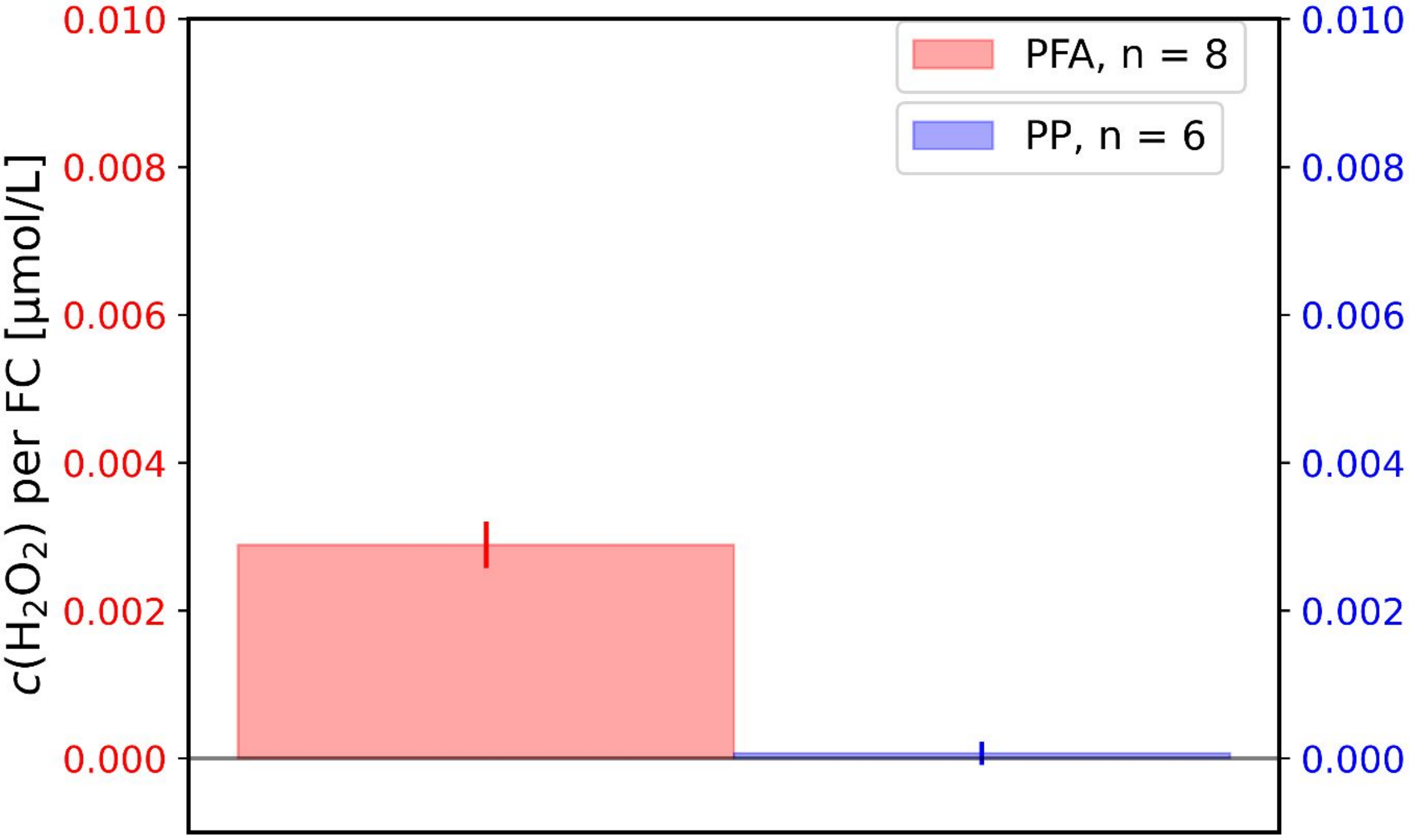

S1_1: Role of the test tube material: Comparison of H_2O_2 yield in PFA and PP test tubes for -15 °C bath temperature, ultrapure water (errors are represented as arithmetic mean deviation).

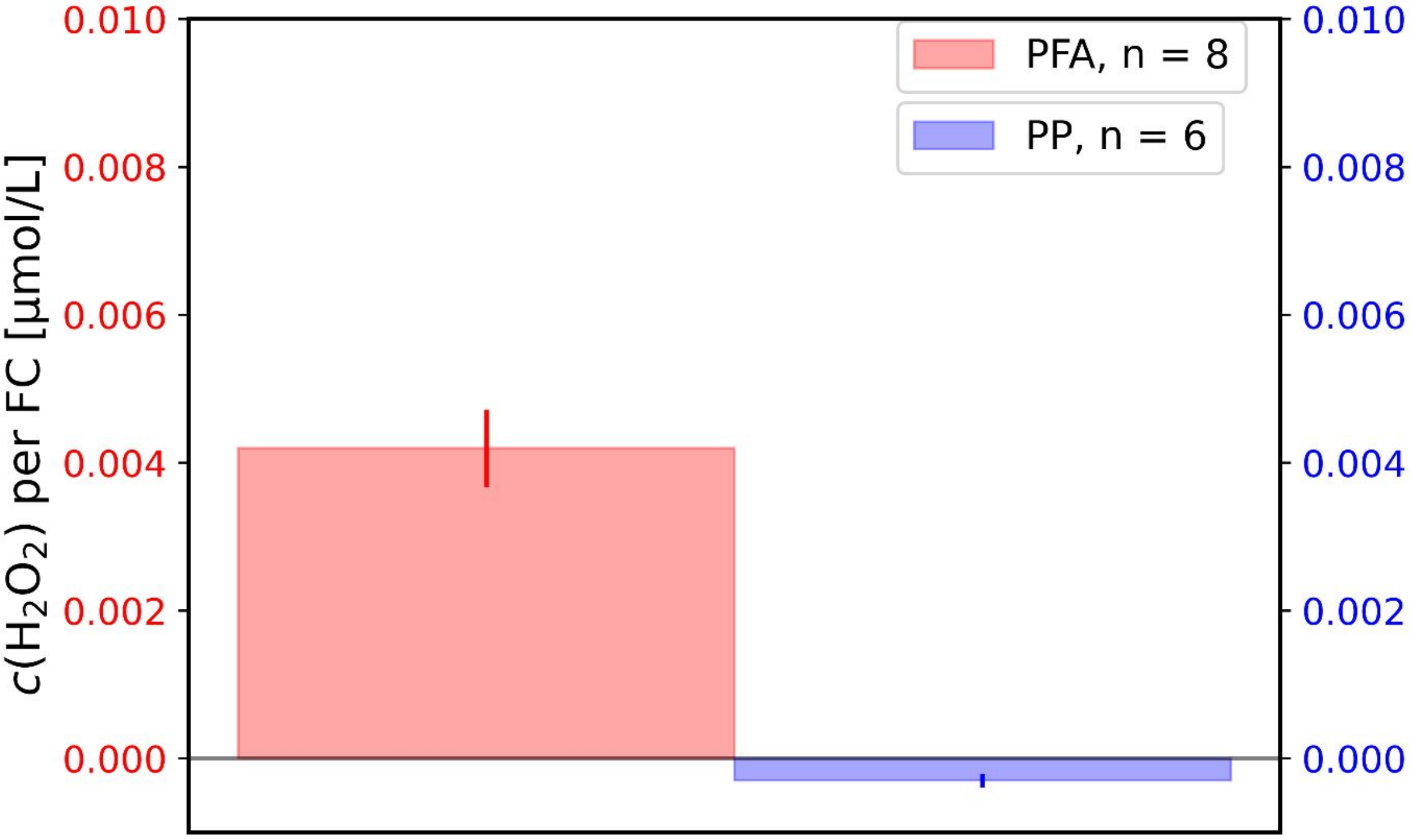

S1_2: Role of the test tube material: Comparison of H_2O_2 yield in PFA and PP test tubes for -20 °C bath temperature, NaCl 10^{-4} M (errors are represented as arithmetic mean deviation).

II. H_2O_2 variation from freezing and/or thawing

To assess potential losses of H_2O_2 during freeze-thaw cycling, control experiments were performed using aqueous solutions with known H_2O_2 concentrations subjected to repeated freezing and thawing in PP and PFA test tubes. PP test tubes were used as controls since they show negligible H_2O_2 formation. In the PFA experiments, 10^{-1} M NaCl solution was used, where H_2O_2 formation is substantially reduced. The resulting concentration changes were within experimental uncertainty, indicating negligible systematic loss under the applied conditions.

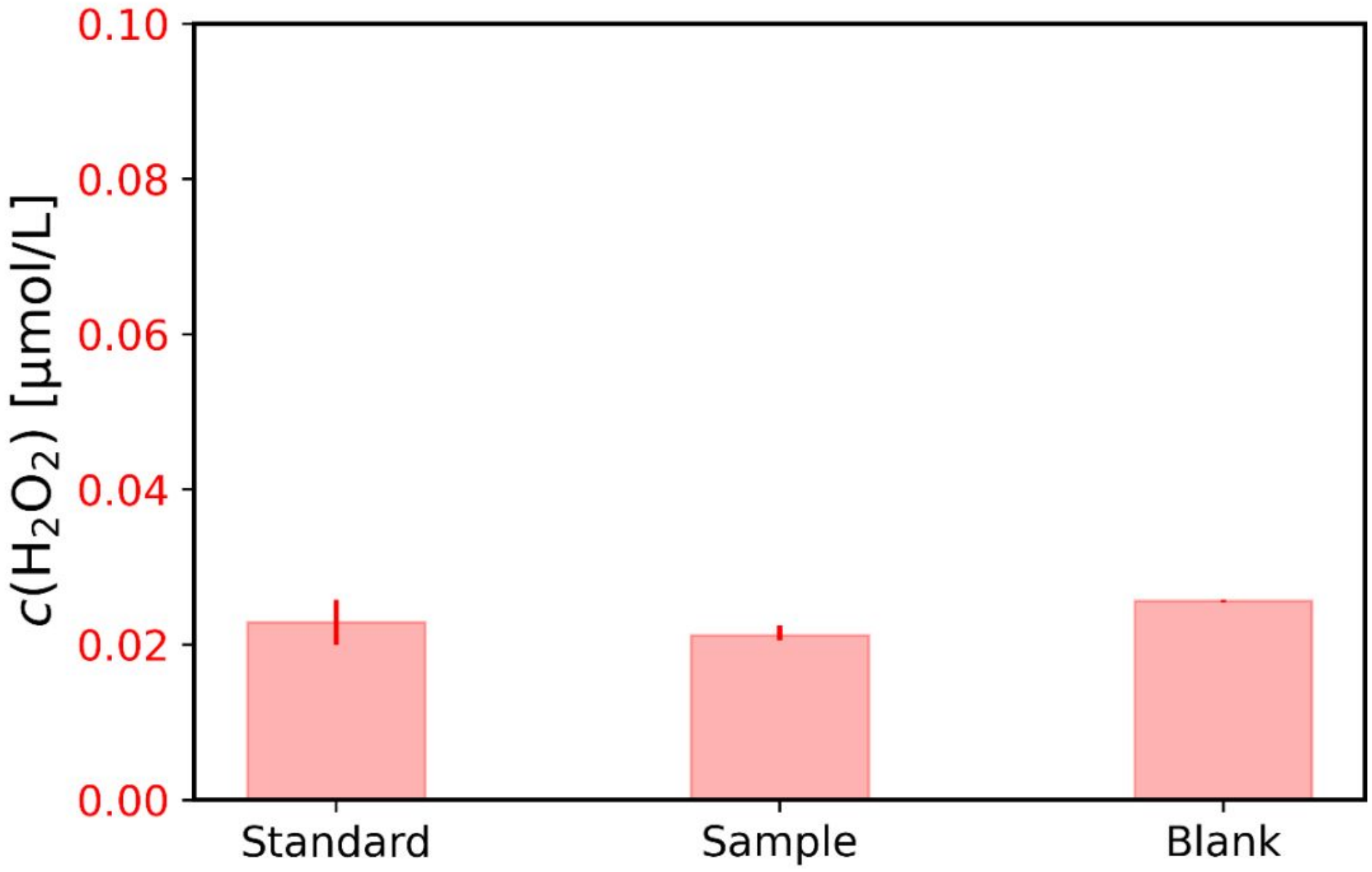

S1_3: H_2O_2 variation during freezing and/or thawing of 0.025 µmol/L H_2O_2 solution in PP (errors are represented as span, i.e., deviation between the arithmetic mean and the maximum and minimum values).

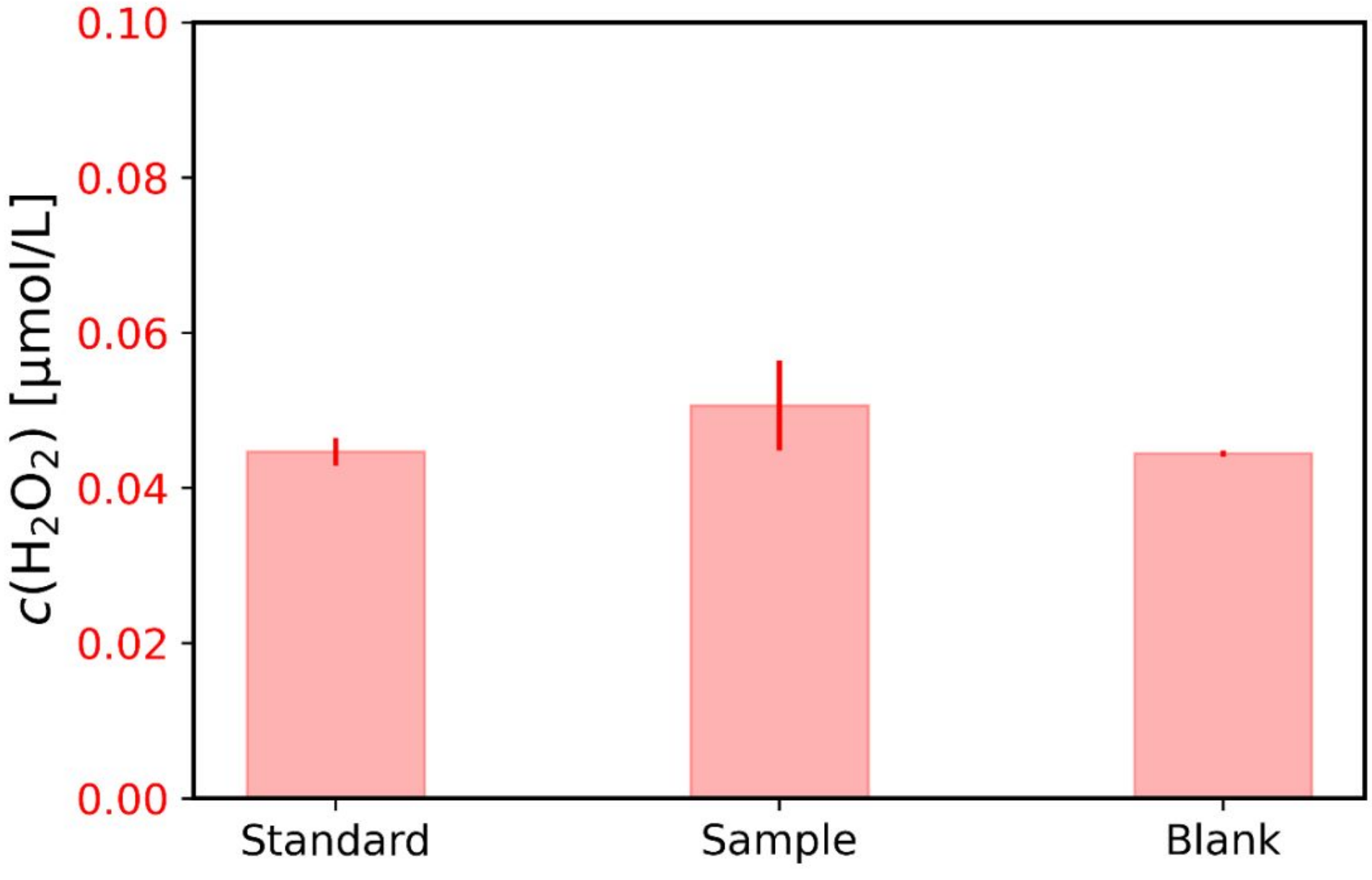

S1_4: H_2O_2 variation during freezing and/or thawing in PFA of 0.050 µmol/L H_2O_2 + 10^{-4} M NaCl solution (errors are represented as span, i.e., deviation between the arithmetic mean and the maximum and minimum values).

For PP, the difference between the samples and corresponding blanks was approximately -0.0006 µmol/L per FC for an initial H_2O_2 concentration of 0.025 µmol/L (n(samples)=3, n(blanks)=2; Fig. S1_3). This value is comparable to the small concentration differences observed under oxygen-depleted conditions in PFA test tubes and remains close to the experimental uncertainty of the method. Similar observations were obtained for the 10^{-1} M NaCl experiments (initial H_2O_2 concentration 0.050 µmol/L), where no statistically significant decrease relative to controls was observed; instead, a change of approximately +0.0006 µmol/L per FC in respect to the blank samples (n(samples)=3, n(blanks)=2; Fig. S1_4) was found.

Overall, these results suggest that any phase transition-induced H_2O_2 loss, if present, is small compared to the observed H_2O_2 formation in PFA systems and does not significantly affect the interpretation of the reported trends. Nevertheless, a minor underestimation of the absolute H_2O_2 yield cannot be excluded.

III. Dependency of H_2O_2 yield per FC on the ethanol bath temperature

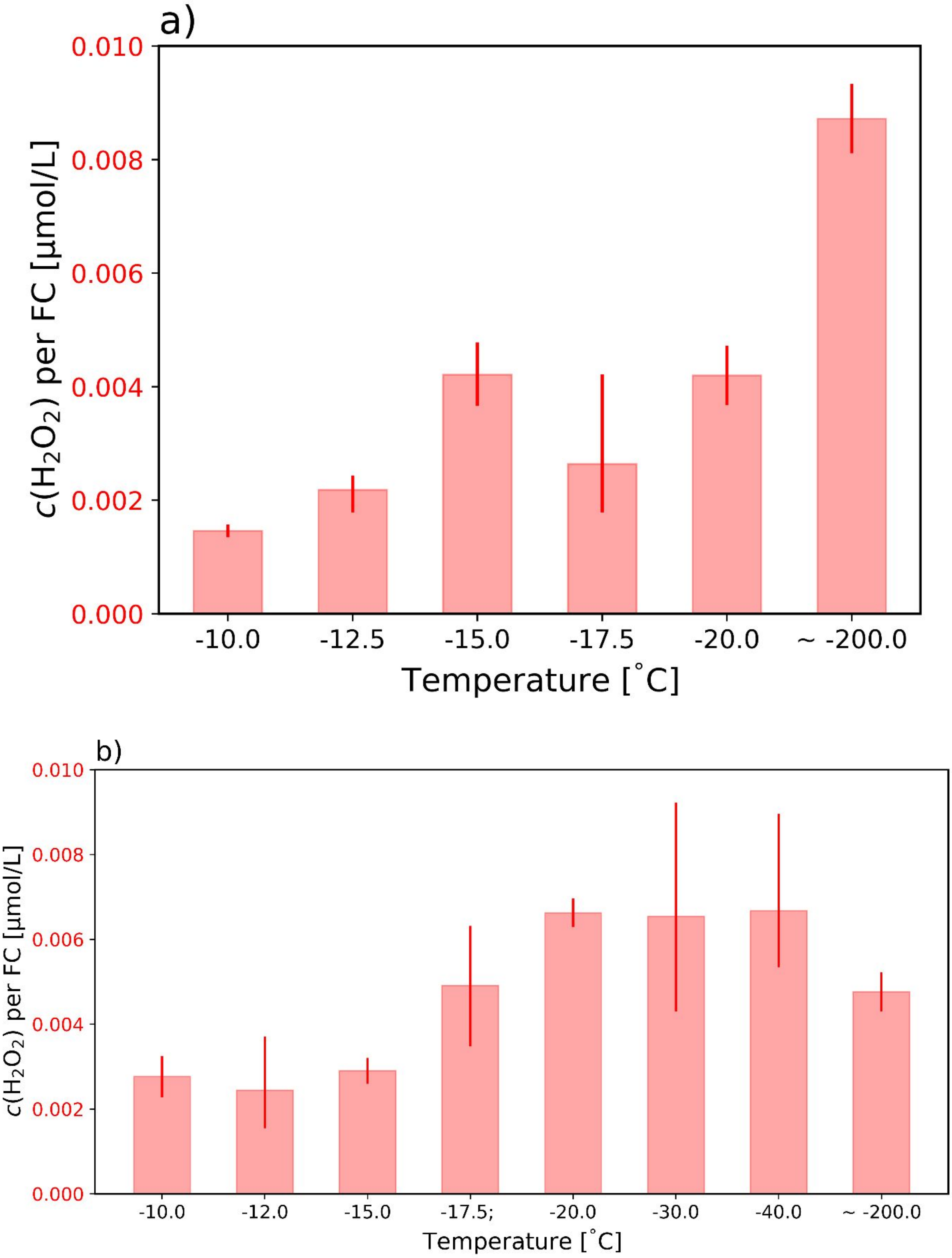

S1_5: Dependency of blank-corrected H_2O_2 yield per FC for (a) 10^{-4} M NaCl solution and (b) ultrapure water frozen at temperatures down to ~ -200 °C (errors are represented as arithmetic mean deviation; for n > 3, errors are represented as standard error of the mean; otherwise as span, i.e., deviation between arithmetic mean and maximum and minimum values). The number of Samples (n(Samples)) is given in Table 1.

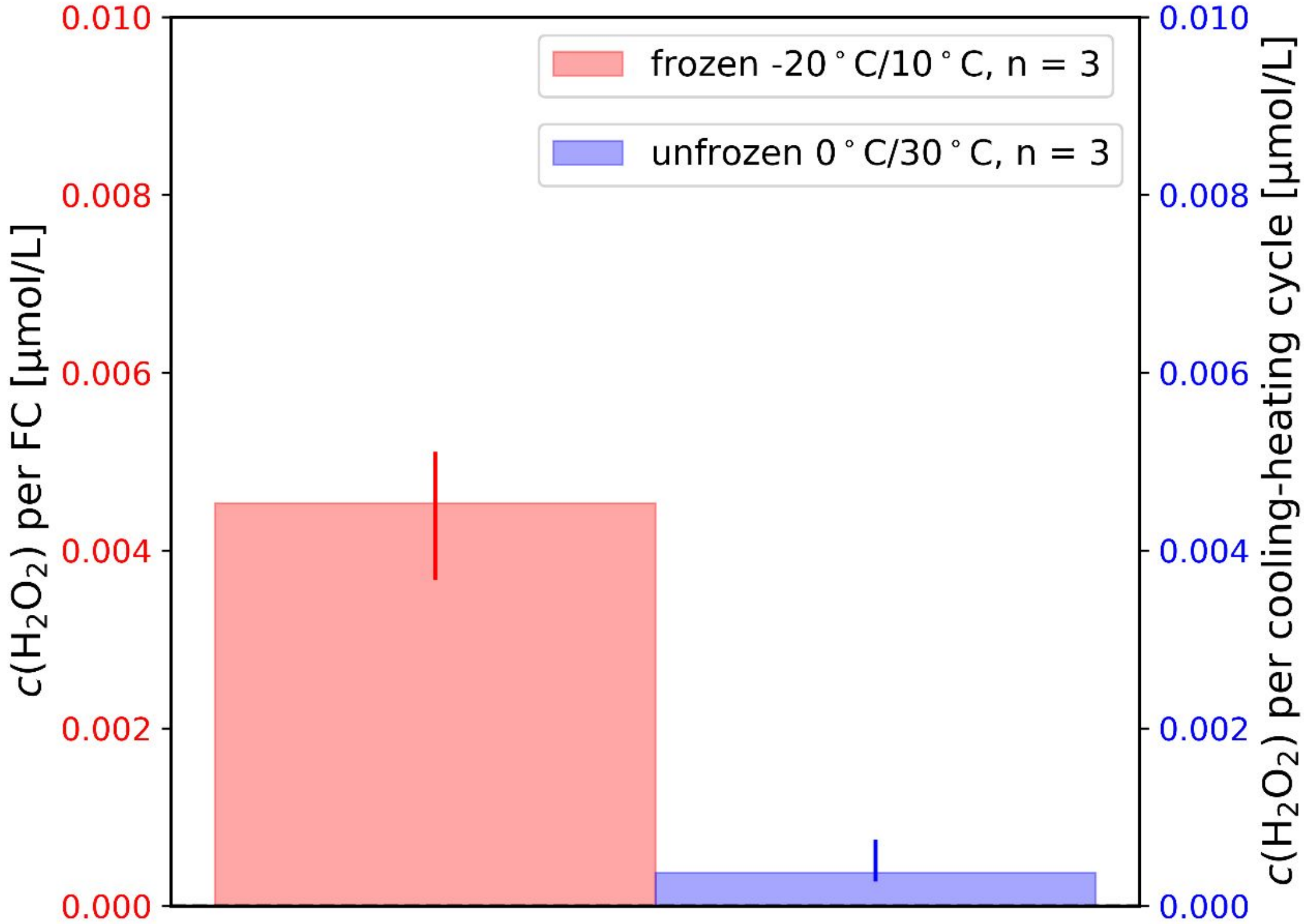

S1_6: Comparison of blank-corrected H_2O_2 yield for ultrapure water frozen at -20 °C, melted at 10 °C and ultrapure water unfrozen cooled for 10 min at 0 °C, then heated for 10 min at ~30 °C (errors are represented by the span (deviation between the arithmetic mean value and max. and min. value)).

Section S2 – Additional Setup Information

I. Bulk freezing experiments

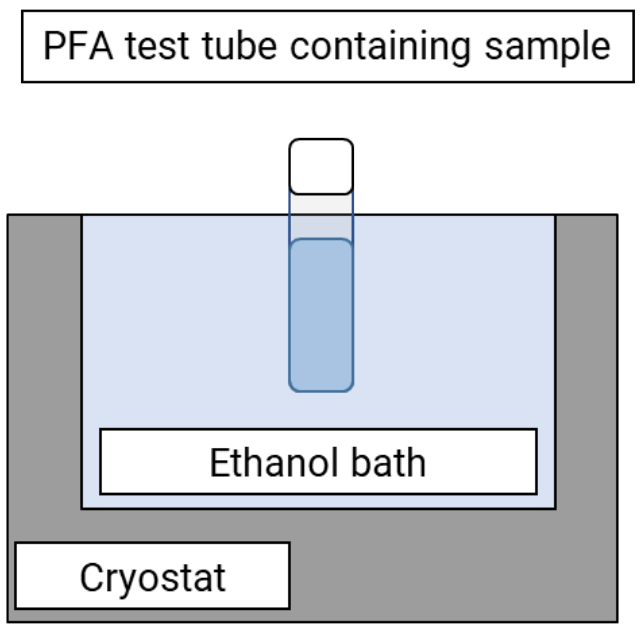

S2_1: Experimental setup for freezing aqueous systems.

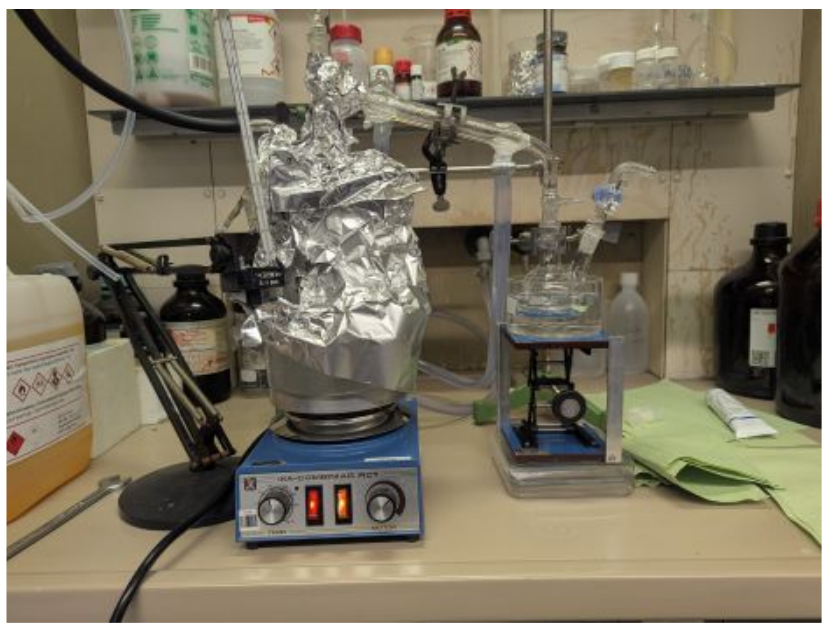

S2_2: Distillation to deoxygenate water.

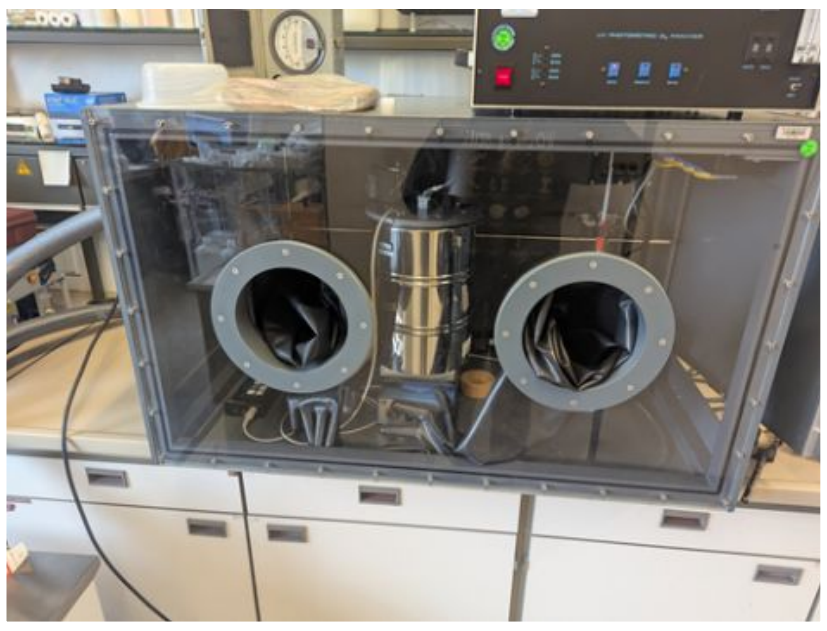

S2_3: Glove box with EtOH bath and ozone monitor.

II. Titled plate setup

For charge measurement, DI water droplet (35 µL) was dispensed on the polymer surface on a tilted plate (50˚)[33]. An electrode was placed near the surface at a sliding distance of 6 cm to measure charge. The electrode was connected to a current amplifier (DDPCA-300, FEMTO) and a data acquisition system (DAQ, USB-6366, NI) to record the current signal when a droplet contacted the electrode. The current signal was integrated to estimate the amount of charge. The time interval between subsequent droplets was taken as 8 sec. All surfaces were neutralized with an ion blower (Simco-Ion Aerostat) for at least 5 minutes prior to the experiments to remove residual charge.

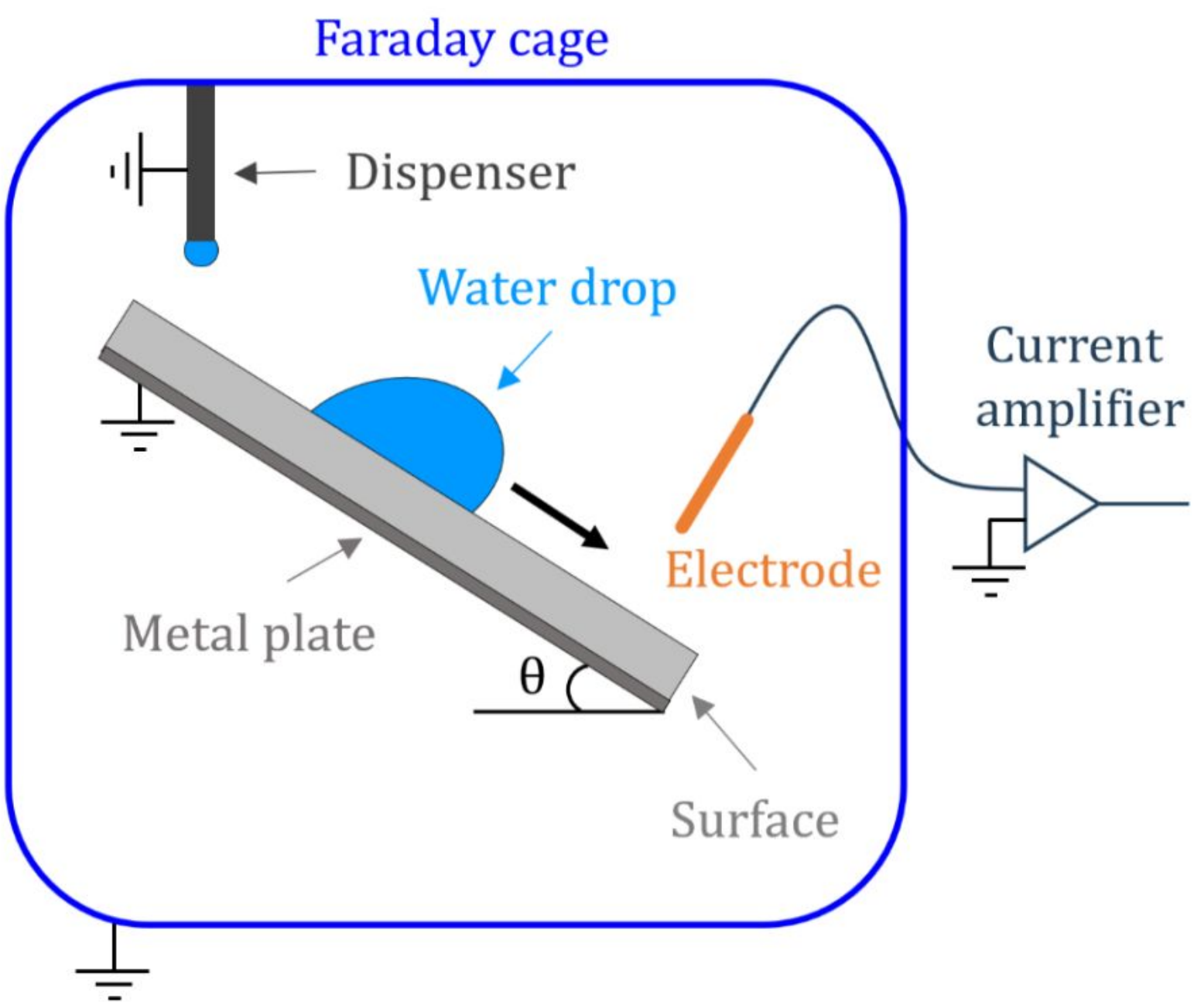

Fig. 2_4: Tilted plate setup[33]: Measurement of droplet charge by sliding Milli-Q water over different surfaces (PFA and PP), ϴ = 50˚.

Section S3 – Measurement of H_2O_2

The concentration of H_2O_2 in all aqueous systems was determined after the freezing and thawing cycles using the AL2021 AEROLASER H2O2 Monitor from "Gesellschaft für Gasanalytik" in liquid phase measurement mode (LOD ≥ 0.1 µg/L). The detection is based on the peroxidase-catalyzed reaction of p-hydroxyphenylacetic acid with peroxides to 2,2′-Bis(carboxymethyl)-4,4′-dihydroxy-1,1′-biphenyl, a fluorescent dimer ($\lambda_{exc.}$ = 326 nm; $\lambda_{em.}$ = 400-420 nm). To distinguish between H_2O_2 and organic peroxides, a second channel is used, in which H_2O_2 is destroyed by catalase prior to fluorescent detection, and the amount of H_2O_2 is inferred from the difference between the channel with and the channel without catalase (see S3_1). The device has always been internally calibrated with a 0.20 µmol/L H_2O_2 Standard, produced by diluting a 30% hydrogen peroxide solution, and an external calibration with 0.20 µmol/L, 0.10 µmol/L, and 0.050 µmol/L reference standards was performed to calculate the H_2O_2 concentration of the samples. The ultrapure water was set to zero.

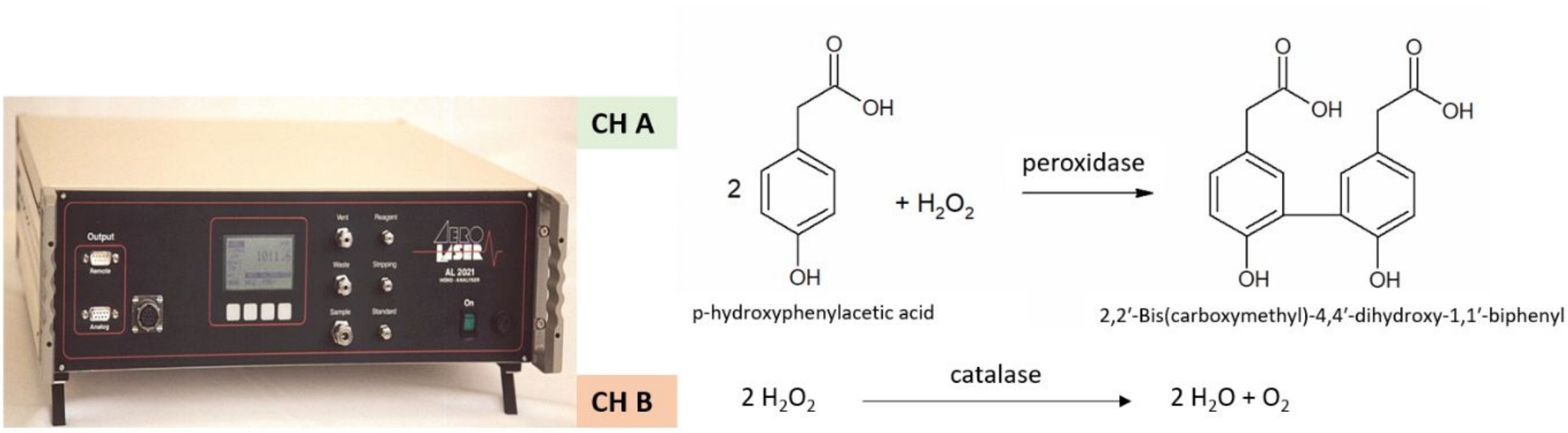

S3_1: AL2021 AEROLASER H2O2 Monitor from Gesellschaft für Gasanalytik, Garmisch-Patenkirchen, Germany and detection reaction for H_2O_2 using p-hyroxyphenylacetic acid.

Section S4 – Number of samples (n)

Table 1: n(Samples) for Figures 3 and S1_5.

Figure(s)	c(NaCl) [mol/L]	n(Samples)
3a) + 3b)	~0	10
	10^{-5}	3
	10^{-4}	7
	10^{-3}	3
	10^{-2}	3
	10^{-1}	3
3c)	~0	14
	10^{-5}	7
	10^{-4}	13
	10^{-3}	3
	10^{-2}	3
	10^{-1}	3
**Figure(s)**	**Temperature [°C]**	**n(Samples)**
S1_5a)	-10.0	6
	-12.5	3

	-15.0	3
	-17.5	3
	-20.0	8
	~ -200	13
S1_5b)	-10.0	6
	-12.0	3
	-15.0	8
	-17.5	3
	-20.0	10
	-30.0	3
	-40.0	3
	~ -200	14
